# Effects of Heat-Induced Oxidative Stress and Astaxanthin on the *NF-kB*, *NFE2L2* and *PPARα* Transcription Factors and Cytoprotective Capacity in the Thymus of Broilers

**DOI:** 10.3390/cimb46080544

**Published:** 2024-08-22

**Authors:** Donna Lee Kuehu, Yuanyuan Fu, Masaki Nasu, Hua Yang, Vedbar S. Khadka, Youping Deng

**Affiliations:** 1 Bioinformatics Core, Department of Quantitative Health Sciences, John A. Burns School of Medicine, University of Hawaii, Honolulu, HI 96813, USA; dkuehu@hawaii.edu (D.L.K.); fuy@hawaii.edu (Y.F.); mnasu@hawaii.edu (M.N.); yanghua@hawaii.edu (H.Y.); vedbar@hawaii.edu (V.S.K.); 2 Department of Molecular Biosciences and Bioengineering, College of Tropical Agriculture and Human Resources, University of Hawaii at Manoa, Honolulu, HI 96822, USA

**Keywords:** oxidative stress, astaxanthin, *NF-kB*, *NFE2L2*, cytoprotective capacity, thymus, broilers

## Abstract

The thymus, a central lymphoid organ in animals, serves as the site for T cell development, differentiation and maturation, vital to adaptive immunity. The thymus is critical for maintaining tissue homeostasis to protect against tumors and tissue damage. An overactive or prolonged immune response can lead to oxidative stress from increased production of reactive oxygen species. Heat stress induces oxidative stress and overwhelms the natural antioxidant defense mechanisms. This study’s objectives were to investigate the protective properties of astaxanthin against heat-induced oxidative stress and apoptosis in the chicken thymus, by comparing the growth performance and gene signaling pathways among three groups: thermal neutral, heat stress, and heat stress with astaxanthin. The thermal neutral temperature was 21–22 °C, and the heat stress temperature was 32–35 °C. Both heat stress groups experienced reduced growth performance, while the astaxanthin-treated group showed a slightly lesser decline. The inflammatory response and antioxidant defense system were activated by the upregulation of the *NF-kB*, *NFE2L2*, *PPARα*, cytoprotective capacity, and apoptotic gene pathways during heat stress compared to the thermal neutral group. However, expression levels showed no significant differences between the thermal neutral and heat stress with antioxidant groups, suggesting that astaxanthin may mitigate inflammation and oxidative stress damage.

## 1. Introduction

The thymus is a crucial central lymphoid organ in animals, playing an integral role in the immune response by serving as the site for T cell development, differentiation, and maturation [1,2,3]. These mature T cells subsequently colonize secondary lymphoid organs to combat invading pathogens [4]. Beyond its role in adaptive immunity, the thymus is also a critical mediator of innate immune responses, providing protection against tumors, pathogens, and tissue damage [5]. The integrity of the thymus is essential for maintaining tissue homeostasis and a fully functional immune system. Thymic injury can lead to immune impairment, resulting in significant consequences due to the development of an immature immune system, which can leave the organism immunocompromised [6,7,8].

Stress represents a physiological and biochemical defense mechanism through which the body responds to adverse environmental effects. This response helps the organism adapt to its environment and maintain internal equilibrium [9]. While moderate stress can enhance immunity, excessive stress can negatively impact growth, development, and production performance in animals. Importantly, it can also lead to immune suppression, increasing susceptibility to diseases and potentially resulting in death [10]. Persistent oxidative stress (OS), in particular, impairs immune function through mechanisms such as cellular DNA damage and biomolecule fragmentation [11,12,13]. Normally, the body’s oxidation-antioxidant system maintains a dynamic balance, but OS occurs when the antioxidant (AOX) defenses are overwhelmed [14,15]. This condition can lead to slower growth rates, decreased feed conversion efficiency, reduced production performance, and in severe cases, significant economic losses in industries like poultry farming [16,17]. OS initiates various signaling pathways and inflammatory responses, further compounding its impact on health [18].

The nuclear factor kappa-light-chain-enhancer of activated B cells (*NF-kB*) signaling pathway is a key mediator of OS-induced inflammation [19,20,21,22]. Activation of *NF-kB* leads to the production of inflammatory cytokines, which are part of the body’s response to harmful stimuli. Inflammation, closely linked to the immune system, is a pathological response to such stimuli [23]. Additionally, the transcription factor nuclear factor erythroid 2-like-2 (*NFE2L2/NRF2*) plays a significant role in cytoprotection by stimulating the expression of AOX and detoxifying enzymes, including NAD(P)H: quinone oxidoreductase-1 (NQO-1), glutathione S-transferase (GST), and heme-oxygenase-1 (HO-1) [24]. The AOX defense system comprises both enzymatic AOXs, such as superoxide dismutase (*SOD*), peroxidase (*PRDX*), catalase (*CAT*), and glutathione peroxidase (*GPX1*) and non-enzymatic AOXs, including vitamin E, carotenoids, and vitamin C. An imbalance between oxidative and AOX systems can lead to excessive reactive oxygen species (ROS) and reactive nitrogen species (RNS) accumulation, resulting in the destruction of lipids, proteins, and nucleic acids, ultimately causing cellular damage and cell death. OS is thus implicated in a variety of pathological conditions, including inflammatory reactions and tumor development [25,26,27]. Peroxisome proliferator-activated receptor alpha (*PPARα*) is a nuclear receptor that regulates the genes involved in lipid metabolism, fatty acid oxidation, and inflammation and plays a crucial role in maintaining metabolic homeostasis. PPARα has been shown to produce significant anti-inflammatory effects [28,29] and inhibit the activation of *NF-kB* [30].

Heat-induced OS poses a particular challenge in animals, affecting their ability to metabolize carotenoids, which necessitates dietary supplementation [31]. Astaxanthin (AST), a potent lipid-soluble AOX from the carotenoid family, has demonstrated exceptional free radical scavenging and anti-inflammatory properties [32,33,34,35]. Its unique structure, featuring hydroxyl and keto groups on each ionone ring, enhances its AOX activity, thereby protecting cellular membranes from oxidation [36]. Despite its presence in aquatic organisms and birds, AST cannot be synthesized by animals and must be obtained from dietary sources such as green algae, red yeast, and crustacean byproducts, with *Haematococcus pluvialis* being a particularly rich source [37,38]. The carotenoid antioxidant value of powder AST is described as the Oxygen Radical Absorbance Capacity (ORAC) and expressed in micromoles of Trolox equivalents per 100 g (μmol TE/100 g), a vitamin E analog. AST has been shown to have 6000 times the AOX capacity of vitamin C, 800 times that of CoQ10, and 550 times that of vitamin E [39]. Supplementation with AST has shown numerous health benefits, including anti-inflammatory, immunomodulatory, cardiovascular, neuroprotective, and anticancer effects [40]. In poultry farming, particularly under heat-stress conditions, AST holds promise as a natural AOX feed supplement, potentially mitigating the adverse effects of heat stress and improving overall health and productivity. 

The main objectives of the present study were to investigate the protective properties of AST against heat-induced OS and apoptosis in the chicken thymus. Our research question is as follows: “How does heat-induced OS affect the *NF-kB*, *NFE2L2*, *PPARα*, cytoprotective capacity genes and apoptotic pathways in the broiler thymus, and can AST help mitigate the stress?” We hypothesize that heat-induced OS in the broiler thymus correlates with poor growth performance, disrupting the physiological and biochemical defense mechanisms, and that *H. pluvialis*-derived AST dietary supplementation may play a role in mitigating the effects through modulation of transcription pathways.

## 2. Materials and Methods

### 2.1. Ethics Statement

The animal protocol (Protocol No. 17-2605) used in this study was approved by the University of Hawaii Institutional Animal Care and Use Committee (IACUC). Animals were raised under animal welfare guidelines and euthanized in accordance with humane protocols in preparation for necropsy.

### 2.2. Experimental Animal Design

Cobb 500 unsexed broiler chicks were obtained from Asagi Hatchery (Honolulu, HI, USA). Asagi Hatchery is a local commercial producer that sells newly hatched chicks for commercial or research purposes; we have permission to utilize the resource they provide without a need for written consent, but we acknowledge them as a source. Several mitigation strategies were simultaneously tested in parallel, and the findings were reported separately based on treatment. In this trial, the feed additive treatment is AST. One-day-old chicks were weighed, wing tagged, and then separated into groups of 4 birds in each pen, (*n* = 60). The pens were established on concrete flooring, prepared with pine shavings, and stocked with water bottles and open feeders. Animals were provided a normal starter feed from 0 to 21 days, and a normal finisher feed on 22–42 days with free access to feed and water. Nutritional compositions of the supplemented diets are listed in Appendix A (Table A1). The animals were reared under two temperature conditions: thermal neutral (TN) (*n* = 24) at 21–22 °C and 50% RH, and heat stress (HS) (*n* = 36) at 32–35 °C and 42–50% RH. In the first 21 days, all birds were raised under the TN temperature conditions and fed the same diet. In the last 21 days, the two groups raised under the HS temperature conditions were further divided into two dietary regimens, i.e., basal diet HS (*n* = 18, treatment 1), and basal diet with 1.33 mg/kg AX supplement (HSAX) (*n* = 18, treatment 2). The light cycle was set at a 1:23 dark:light cycle throughout the trial. Six broiler hens were randomly selected from each group and euthanized on day 42, and thymus tissue samples were collected at necropsy. 

### 2.3. Astaxanthin-Rich Dietary Supplement

The diet was supplemented with P25HB provided by AstaReal^®^, Inc. (Burlington, NJ, USA). PH25B contains 2.5% (*w*/*w*) dried *Haematococcus pluvialis* algae as well as other components including modified starch, gum Arabic, mixed tocopherols, L-ascorbyl palmitate, silicon dioxide, xanthan gum, γ–cyclodextrin, polysorbate 80, rosemary extract and ferulic acid. A comparable nutritional composition of *H. pluvialis* algae is listed in Appendix A (Table A2). The natural forms of astaxanthin mainly comprise mono-esterified, followed by di-esterified and free forms 3,3′-dihydroxy-β, β-carotene-4 and 4′-dione (C_40_H_52_0_4_ free form). Fuji Health Sciences, Inc. (AstaReal Inc., Moses Lake, WA, USA), AstaReal ORAC value is reported at 2,822,200 μmol TE/100 g, supported by Non-US Gov’t: Brunswick Laboratories Test Report Batch No. B-10267b-2010.

### 2.4. Growth Indicators

Weekly feed intake was recorded, and the average daily feed intake (ADFI), average daily gain ratio (ADG), and feed conversion ratio (FCR) were calculated. The body weight (BW) of each bird was recorded using a Mettler Toledo scale before heat stress treatment and at the end of the heat treatment. 

### 2.5. Tissue Sample Collection

Immediately after euthanizing, thymus tissue samples were collected from 6 randomly selected birds from each group, which were snapped-frozen in liquid nitrogen and stored at −80 °C.

### 2.6. Total RNA Extraction and cDNA Preparation

Total RNA was isolated from frozen tissues (50–100 mg) using TRIzol reagent (Invitrogen, Carlsbad, CA, USA) according to the manufacturer’s instructions. The concentration of total RNA was determined using a NanoPhotometer^®^ P330 (IMPLEN, Los Angeles, CA, USA). Complementary DNA (cDNA) was synthesized from 1 µg of total RNA (20 µL reaction of RT mixture) using a High-Capacity cDNA Reverse Transcription Kit (Applied Biosystems, Foster City, CA, USA) and further diluted with nuclease-free water (1:25) for the qPCR reaction outlined below.

### 2.7. Bioinformatics: Genome Assembly and Gene Primer Design

The National Center for Biotechnology Information (NCBI) genome browser was used to search and compile genes for *Gallus gallus domesticus* related to heat stress, oxidative stress, cytoprotective, epithelial integrity, transcription factors and housekeeping genes. The NCBI-Basic Local Alignment Search Tool (BLAST) was used to design primers for polymerase chain reaction (PCR) from the accession numbers obtained from the list of genes presented in Appendix A (Table A3). The primer parameters were set for a PCR product size between a minimum of 100 and maximum of 250 for 5 primers to return. The primer melting temperatures were set for a minimum of 55 °C, optimum of 57 °C, and maximum of 60 °C with a maximum Tm difference of 3 °C. The exon junction span was set so that the primers must span an exon–exon junction. The organism specified was *Gallus gallus* (taxid 9031). The forward and reverse primer sequences (5′→ 3′) were then submitted to Integrated DNA Technologies (Coralville, IA, USA) for synthesis. 

### 2.8. Quantitative Real-Time RT-PCR (qPCR)

The qPCR was performed using PerfeCTa SYBR Green FastMix (Quantabio, Beverly, MA, USA) on a Q—qPCR instrument (Quantabio, Beverly, MA, USA). The qPCR reaction mixture consisted of 2 µL of cDNA, 10 µL PerfeCTa SYBR Green FastMix, 1 µL of each forward and reverse primer (5 µmol concentration), and 6 µL of sterile deionized water to make a final reaction mixture of 20 µL. Specific primer pairs for the detection of each gene were designed using the NCBI Primer-Blast tool. The qPCR reaction was carried out following the standard cycling mode. The amplification conditions were 50 °C for 2 min (hold) and then 95 °C for 2 min (hold) followed by 40 repeat cycles of 95 °C for 15 s (denaturation), 60 °C for 15 s (annealing), and 72 °C for 1 min (extension). A melting curve was also generated to confirm SYBR Green-based objective amplicon, and further qPCR products were confirmed using 2% agarose gel electrophoresis. Three house-keeping genes, glyceraldehyde 3-phosphate dehydrogenase (*GAPDH*), beta-actin (*ACTB*), and TATA-Box Binding Protein (*TBP*), were analyzed in triplicates in each bird to determine the most stable house-keeping gene. Based on the uniformity of the expression level across samples, *ACTB* was chosen as the housekeeping gene. The gene expression level was determined using cycle threshold (Ct) values following the standard curve method after normalization with housekeeping genes. The fold change for each gene was calculated using the 2^−ΔΔCt^ method and presented as mean ± standard error [41]. 

### 2.9. Gene Ontology

Significantly differentially expressed genes identified from the qPCR procedure were searched in the *Ensembl* genome database for chicken (GRCg6a) species to obtain gene ontology (GO) information (https://www.ensembl.org/index.html, accessed on 2 April 2024) [42]. The GO information included the cellular component, molecular function and biological process of these genes identified with ENSGAL Transcript IDs.

### 2.10. Statistical Analysis

Statistical analysis was performed using the Kruskal–Wallis rank sum test with statistical significance set at *p* < 0.05, followed by the Dunn post hoc test for comparison between three groups: TN, HS, and HSAX, and the *p*-value adjusted using the Bonferroni method. Growth performance measurements were calculated based on the data collected at the end of the 42-day trial period. Analysis was conducted using the R open source program, libraries ‘FSA’, ‘dunn.test’, and ‘gplots’, R Core Team (2023) (https://www.R-project.org/ accessed on 2 April 2024) [43].

## 3. Results

### 3.1. Growth Performance

**Figure 1:**
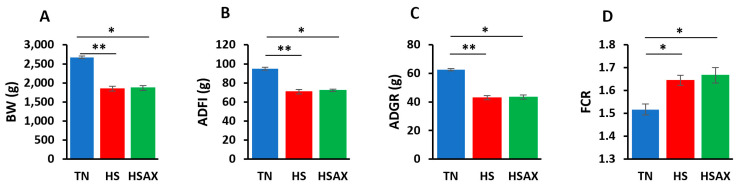
Growth performance indicators of Cobb 500 broilers. Three groups were compared: TN, HS, and HSAX. Dunn post hoc test for pair-wise comparison of statistical significance represented the follows: (**A**) BW (g), TN vs. HS (*p* = 0.010); TN vs. HSAX (*p* = 0.028); (**B**) ADFI(g), TN vs. HS (*p* = 0.005); TN vs. HSAX (*p* = 0.050); (**C**) ADG(g), TN vs. HS (*p* = 0.010); TN vs. HSAX (*p* = 0.0278); and (**D**) FCR, TN vs. HS (*p* = 0.023); TN vs. HSAX (*p* = 0.012). * Differences between treatments significant at *p* < 0.05; ** differences between treatments significant at *p* < 0.01.

At the end of the 42-day poultry trial, the TN group growth performance indicators were found to be significantly higher for BW, ADFI, and ADG compared to the HS and HSAX groups, as established through a Kruskal–Wallis test to evaluate differences among the three groups (*p* < 0.01). Although the results clearly showed negative impacts of heat on the HS and HSAX groups, when performing the post hoc pairwise comparisons using Dunn’s test and applying the Bonferroni correction to control increased risk of error due to multiple comparisons, the negative impacts were found to be more significant in the HS group compared to the TN (*p* < 0.01), whereas the negative impact was slightly less in the HSAX group (*p* < 0.05). In addition, the FCR showed that the TN group was significantly lower in feed conversion, requiring less feed to maintain body weight compared to the HS and *p* < 0.05 (Table 1 and Figure 1). 

### 3.2. Quantitative Real-Time RT-PCR (qPCR) Gene Expression

**Figure 2:**
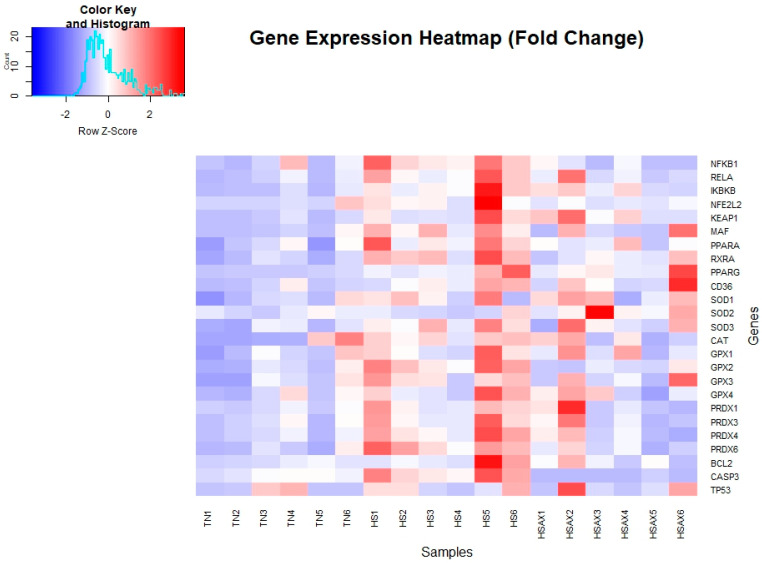
Gene expression fold change heatmap for thymus tissue of Cobb 500 broilers. Three groups were compared: TN, HS, and HSAX. The HS group expression values are shown in shades of red to indicate significantly higher expression, whereas blue indicates lower levels of expression in the TN and HSAX groups.

**Figure 3:**
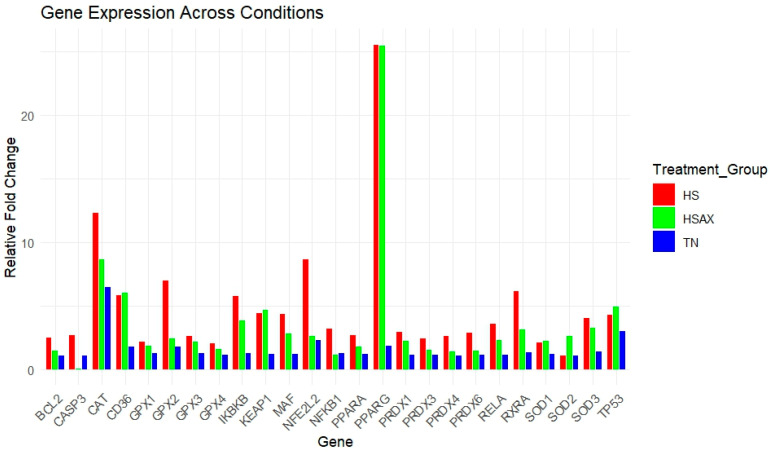
Gene expression fold change across conditions for thymus samples of Cobb 500 broilers. Three treatment groups were expressed: TN (blue), HS (red), and HSAX (green).

For gene expression studies of the thymus, three genes were considered for housekeeping genes: *GAPDH*, *ACTB*, and *TBP*. *ACTB* was selected for its high and relatively stable expression under the experimental conditions performed, making it a reliable reference for normalizing the gene expression data collected. Kruskal–Wallis rank sum test with statistical significance set at *p* < 0.05 was conducted comparing the three groups: TN, HS, and HSAX (Table 2). A gene expression heat map of all samples profiling the visual aspect of the fold change data is displayed in Figure 2. A histogram of gene expression of relative fold change levels across all genes provides a summary comparison between the treatment groups (Figure 3).

#### 3.2.1. NF-kB Transcription Signaling Pathway Genes

**Figure 4:**
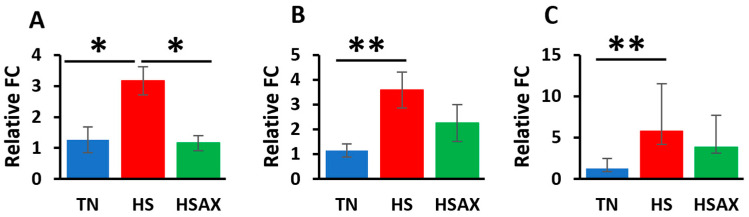
Effects of heat stress and astaxanthin treatment in the *NF-kB* transcription factor signaling pathway in the thymus tissue of Cobb 500 broilers. Three groups were compared: TN, HS, and HSAX. Dunn post hoc test for pair-wise comparison of statistical significance represented the following: (**A**) *NFKB1*, TN vs. HS (*p* = 0.028), HS vs. HSAX (*p* = 0.028); (**B**) *RELA*, TN vs. HS (*p* = 0.009); (**C**) *IKBKB*, TN vs. HS (*p* = 0.011). * Differences between treatments significant at *p* < 0.05; ** differences between treatments significant at *p* < 0.01.

The studies showed the impact of HS and AOX treatment expressed through the *NF-kB* transcription factor signaling pathway and pairwise comparisons (Figure 4). *NFKB1* (*p* = 0.028), *NF-kB* subunit (*RELA*) (*p* = 0.009), and inhibitor of nuclear factor kappa B kinase subunit beta *(IKBKB*) (*p* = 0.011) were found to be more upregulated in the HS group in comparison to the TN group. In addition, the expression of *NFKB1* (*p* = 0.028) was found to be more upregulated in the HS group than in the HSAX group.

#### 3.2.2. NFE2L2-Mediated Signaling Pathway Genes

**Figure 5:**
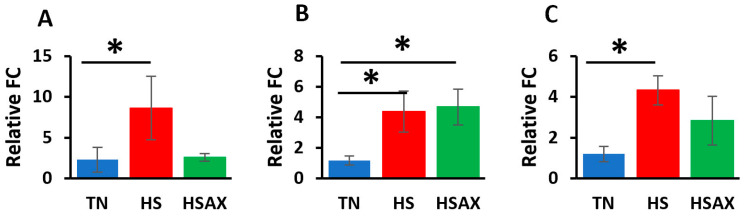
Effects of heat stress and astaxanthin treatment in the *NFE2L2*-mediated signaling pathway in the thymus tissue of Cobb 500 broilers. Three groups were compared: TN, HS, and HSAX. Dunn post hoc test for pair-wise comparison of statistical significance represented the following: (**A**) *NFE2L2*, TN vs. HS (*p* = 0.033); (**B**) *KEAP1*, TN vs. HS (*p* = 0.028), TN vs. HSAX (*p* = 0.028); (**C**) *MAF*, TN vs. HS (*p* = 0.024). * Differences between treatments significant at *p* < 0.05.

The results of the impact of HS on the gene expression of the *NFE2L2*-mediated signaling pathway showed that, compared to the TN group, the HS treatment group demonstrated higher upregulation of *NFE2L2* (*p* = 0.033), kelch-like ECH-associated protein 1 (*KEAP1*) (*p* = 0.028), and musculoaponeurotic fibrosarcoma (*MAF*) (*p* = 0.024) (Figure 5). The expression of *KEAP1* (*p* = 0.028) was also more upregulated in the HSAX group compared to the TN group.

#### 3.2.3. PPARα Signaling Pathway Genes

**Figure 6:**
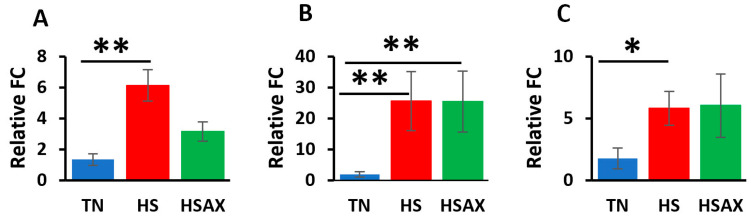
Effects of heat stress and astaxanthin treatment in the *PPARα* signaling pathway in the thymus tissue of Cobb 500 broilers. Three groups were compared: TN, HS, and HSAX. Dunn post hoc test for pair-wise comparison of statistical significance represented the following: (**A**) *RXRA*, TN vs. HS (*p* = 0.003); (**B**) *PPARγ*, TN vs. HS (*p* = 0.011), TN vs. HSAX (*p* = 0.011); (**C**) *CD36*, TN vs. HS (*p* = 0.045). * Differences between treatments significant at *p* < 0.05; ** differences between treatments significant at *p* < 0.01.

Consistent with the findings for the *NF-kB* and *NFE2L2* pathways, the *PPARα* pathway showed similar results with the HS group showing higher upregulation in terms of the gene expressions of retinoid X receptor alpha (*RXRA*) (*p* = 0.003), peroxisome proliferator-activated receptor gamma (*PPARγ*) (*p* = 0.011), and cluster of differentiation 36 (*CD36*) (*p* = 0.045) compared to the TN group (Figure 6). The HSAX group also showed higher upregulation of *PPARγ* (*p* = 0.011) compared to the TN group.

#### 3.2.4. Cytoprotective Capacity Genes

**Figure 7:**
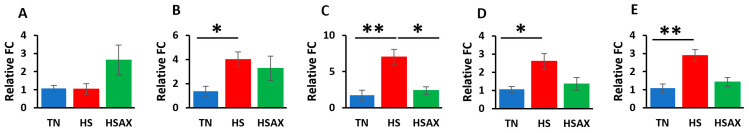
Effects of heat stress and astaxanthin treatment on the cytoprotective capacity gene expression in the thymus tissue of Cobb 500 broilers. Three groups were compared: TN, HS, and HSAX. Dunn post hoc test for pair-wise comparison of statistical significance represented the following: (**A**) *SOD2*, HS vs. HSAX (*p* = 0.069); (**B**) *SOD3*, TN vs. HS (*p* = 0.039); (**C**) *GPX2*, TN vs. HS (*p* = 0.006), HS vs. HSAX (*p* = 0.045); (**D**) *PRDX4*, TN vs. HS (*p* = 0.028); (**E**) *PRDX6*, TN vs. HS (*p* = 0.006). * Differences between treatments significant at *p* < 0.05; ** differences between treatments significant at *p* < 0.01.

Results of the cytoprotective capacity genes provided similar results to those of the previously examined transcription factor pathways where the HS group showed higher upregulation than the TN group for the following expressions: superoxide dismutase 3 (*SOD3*) (*p* = 0.039), glutathione peroxidase 2 (*GPX2*) (*p* = 0.006), peroxiredoxin 4 (*PRDX4*) (*p* = 0.028), and peroxiredoxin 6 (*PRDX6*) (*p* = 0.006) (Figure 7). In addition, the HS group was found to be upregulated higher than the HSAX group in the expression of *GPX2* (*p* = 0.0448). Interestingly, although the Kruskal–Wallis test for *SOD2* was shown to be significant, the post hoc test did not indicate significance in the comparisons between the groups. 

#### 3.2.5. Apoptotic Gene

**Figure 8:**
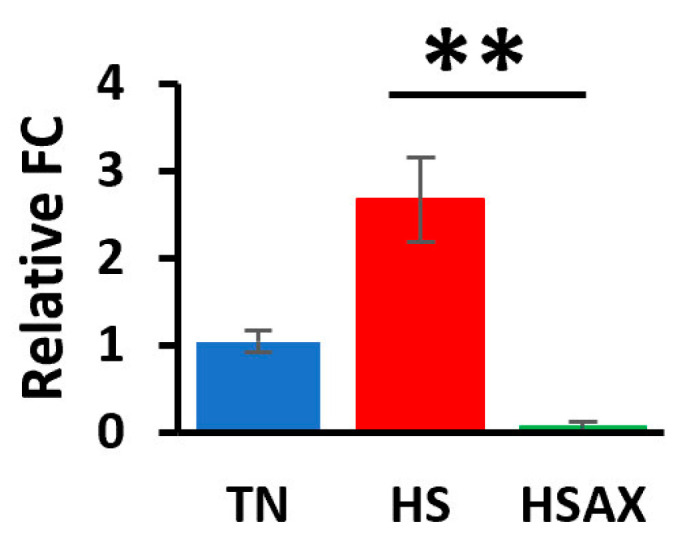
Effects of heat stress and astaxanthin treatment in the apoptotic pathways in the thymus tissue of Cobb 500 broilers. Three groups were compared: TN, HS, and HSAX. Dunn post hoc test for pair-wise comparison of statistical significance represented the following: *CASP3*, HS vs. HSAX (*p* = 0.001). ** differences between treatments significant at *p* < 0.01.

The results of the apoptotic pathway showed a clear indicator in the upregulation of the HS group over the HSAX group in the expression of caspase 3 (*CASP3*) (*p* = 0.001) (Figure 8).

## 4. Discussion

### 4.1. Growth Performance

Growth performance is anticipated to suffer under any stress condition and was confirmed in this study under HS [44]. Reduction in voluntary feed intake, decreased energy availability, altered nutrient digestibility and metabolism, and the breakdown of intestinal epithelial structure and function are some direct negative impacts of heat stress on poultry behavior and physiology [45]. Although the growth performance indexes did not show any significant benefit from an AST supplement based on BW measurements related to feed intake and weight gain, the health of the poultry was further elucidated through the molecular mechanisms of gene expression providing insights affecting the health and wellbeing of the broilers.

### 4.2. Gene Ontology Enrichment and Expression Analysis

#### 4.2.1. *NF-kB* Transcription Signaling Pathway Genes

The *NF-kB* transcription signaling pathway is a critical regulator of immune and inflammatory responses. *NF-kB* can be found throughout the cell in the nucleus, mitochondrion, chromatin and cytoplasm. When not upregulated, *NF-kB* is generally sequestered in the cytoplasm by IkB inhibitor proteins such as *IKBKB*, located in the cytoplasm and cytosol. *RELA* found in the cytoplasm is a key subunit of the *NF-kB* transcription factor complex, needed to form heterodimers of *NF-kB* for translocation into the nucleus upon activation for DNA-binding transcription activity. When activated by stimuli such as stress, free radicals, pathogens and cytokines, IkBs are phosphorylated by a kinase enzyme for ubiquitination and degradation, freeing the *NF-kB* to translocate into the nucleus and promote the transcription of target genes involved in inflammation, immune response, cell proliferation, and survival. The *NF-kB* pathway plays a significant role in responding to cellular stress and homeostasis Appendix A (Table A4) [46].

The outcome of our studies indicates that the upregulation of the HS group over the TN group is consistent with the function of the *NF-kB* complex to respond to cellular stress, and the administration of AST had an attenuating effect. HS exposure has been found to increase *NF-kB* p65 mRNA expression in the spleen of the broiler chicken but inhibited the activation of *NF-kB* p65, reducing the inflammatory response with dietary-seaweed-derived polysaccharides [47]. While there is no significance between the TN and HSAX groups, it indicates that AST has some effect in reducing cellular stress, which coincides with the results in which there is a significant difference between the upregulation of the HS over the HSAX group.

#### 4.2.2. *NFE2L2*-Mediated Signaling Pathway Genes

The *NFE2L2* (*NRF2*) plays a crucial role in cellular defense against OS. *NFE2L2* is found throughout the cell in the nucleus, Golgi apparatus, chromatin, plasma membrane and cytoplasm. Under normal conditions, *NFE2L2* is bound by *KEAP1* in the cytoplasm, and in response to OS or electrophilic stimuli, *NFE2L2* is released to translocate to the nucleus, where it heterodimerizes with small *MAF* proteins. This *NFE2L2-MAF* complex binds to antioxidant response elements (AREs) in the promoters of target genes, leading to transcription of various cytoprotective genes (see Appendix A (Table A4)) [46,48]. 

The results of our study show that the HS group was more upregulated in comparison to the TN group in response to OS throughout the *NFE2L2* signaling pathway. *NFE2L2* is expressed in many tissues and plays a crucial role in combating oxidative stress. Under stressful conditions, *NFE2L2* disassociates from *KEAP1*, leading to increased *NFE2L2* expression levels and enhancing the cell’s antioxidant capacity, which was demonstrated in liver tissue and plasma of broilers subjected to heat stress and supplemented with dietary glutamine [49]. HSAX is upregulated over the TN group in the *KEAP1* gene expression, a critical regulator of the *NFE2L2* signaling pathway, acting as a sensor for OS and controlling the activity of *NFE2L2*, likely a homeostatic feedback mechanism to maintain a balanced redox environment. 

#### 4.2.3. *PPARα* Signaling Pathway Genes

The *PPARα* signaling pathway plays a vital role in lipid metabolism, energy homeostasis and inflammation. Although *PPARα***,** which is predominantly found in the nucleus, has only marginal significance in the upregulation of the HS over the TN group, *RXRA*, *PPARγ* and *CD36* are significantly expressed. *PPARγ* in the HSAX group is also more upregulated in comparison to the TN group. *PPARα* is generally in an inactive state in the nucleus and is activated by fatty acids or specific ligands to form a heterodimer with *RXRA*, and then further undergoes conformational changes for DNA-binding transcription factor activity in the promoter of target genes. Among the target genes is *CD36***,** which facilitates fatty acid uptake and enzymes for lipid metabolism. *PPARγ* can interact with *PPARα* to help regulate lipid metabolism, reduce inflammation and maintain energy homeostasis (see Appendix A (Table A4)) [50].

The results from our study indicate that although there is a subtle response of *PPARα* to heat stress, *PPARγ* appears to be involved in the cellular response to heat stress, suggesting a role in AOX-mediated response to stress. The *PPARα* pathway is involved with lipid metabolism and fatty acid oxidation, where serum malondialdehyde (MDA) has been shown to significantly increase lipid peroxidation due to heat stress in broilers. *Spirulina platensis* supplementation modulated the negative impact of heat stress by reducing the MDA concentration [51]. *RXRA* forms heterodimers with *PPARα* and is essential for transcriptional activity. The upregulation of *CD36* in the HS group compared to the TN group suggests increased lipid metabolism or fatty acid utilization, placing demand on energy due to heat-induced OS.

#### 4.2.4. Cytoprotective Capacity Genes

Cytoprotective capacity genes safeguard cells from various stressors including oxidative damage by enhancing AOX defense mechanisms to promote cell survival. *SOD2* is found in the mitochondrion and responds to OS through the oxidation-reduction process for the removal of superoxide radicals. *SOD3* is found in the extracellular space and plays a crucial role in scavenging superoxide radicals outside the cell. *GPX2* is found in the cytosol, intercellular bridge and mitotic spindle, involved in detoxifying peroxides, such as hydrogen peroxide. *PRDX4* is found in the cytoplasm and endoplasmic reticulum, and *PRDX6* is also found in the cytoplasm, as well as the nucleus. Both peroxiredoxins are involved in scavenging for peroxides, providing peroxide detoxification and protection of cells from OS (see Appendix A (Table A4)) [52,53]. 

The results showed significant upregulation of the HS compared to the TN groups in the expression of *SOD3*, *GPX2*, *PRDX4* and *PRDX6*. There was also significant upregulation of the HS group over the HSAX group for *GPX2*, but only marginally for *PRDX6*. In addition, when analyzing the expression of *SOD3*, there was marginal upregulation of HS over the HSAX group. Related results were produced in a study of meat quality of heat-stressed broilers provided with various supplementation levels of AST for AOX efficacy. In this study, the liver tissue showed an increase in gene expression of the heat-stressed birds for *HSP27* and *HSP70***,** which scavenge for free radicals and assist in proper protein folding, respectively, to reduce oxidative damage. Conversely, AST treatment lowered the gene expression, which may reflect the anti-stress effects of AST and may be due to AOX effects that reduce the adverse impact of heat stress [54]. A separate study on layer hens exposed to heat stress and supplemented with AST resulted in liver tissue showing decreased GPX and enzyme activities of glutathione peroxidase and glutathione S-transferase, suggesting that AST neutralizes free radicals directly or indirectly by removing harmful substrates [55]. On the contrary, a related study conducted on the mRNA expression of ileum tissue of the same subject heat-stressed broilers treated with AST resulted in the HSAX group being significantly upregulated over the HS for *HSF2*, *SOD2,* and *TXN* genes and HSAX upregulated over TN for *GPX3.* The overall outcome was improved epithelial integrity with the upregulation of *LOX*, *CLDN1* and *MUC2* genes in the HSAX group [45]. Similarly, broilers subjected to heat stress resulted in the duodenum tissue mRNA expression of *Nrf2*, *HO-1*, *GPx1* and *GSTT1* being downregulated by HS, and when provided a dietary supplementation of algae-derived polysaccharides, the mRNA expression was upregulated [56]. This indicates a distinct difference between organs with different structures, functions and cell types. Overall, our findings highlight the dynamic regulation of AOX genes in response to heat-induced OS and the potential modulatory effects of AST-AOX supplementation on cellular AOX defense mechanisms.

#### 4.2.5. Apoptotic Pathway Genes

The *CASP3* gene can be found in the cytoplasm and nucleus, where it encodes for a cysteine protease involved in the execution phase of apoptosis. *BCL2* encodes for an anti-apoptotic protein that inhibits cell death, and *TP53* encodes for a tumor suppressor protein involved in cell cycle regulation and apoptosis (see Appendix A (Table A4)) [53,57].

Our findings show significant upregulation of *CASP3* in the HS group over the HSAX group, which suggests the activation of apoptotic pathways, potentially indicating cellular damage or heat-induced OS. Studies using algae-derived polysaccharides to attenuate heat stress in the duodenum and spleen resulted in an increase in the apoptosis rate following heat stress and a decrease in the apoptosis rate after supplementation [47,56]. The lack of significant expression of *BCL2* and *TP53* may suggest that heat stress alone may not induce significant alterations in those apoptotic regulators. However, it appears that the significant reduction in *CASP3* in the HSAX group seems to indicate a protective factor from apoptosis due to the AST supplementation, which may play a role in mitigating the response to heat stress.

## 5. Conclusions

Overall, our experimental findings highlight the dynamic regulation of gene expression in the thymus related to the *NF-kB*, *NFE2L2*, *PPARα*, cytoprotective capacity and apoptotic pathways. Our main objective of the present study was to investigate the protective properties of AST against heat-induced OS and apoptosis in the chicken thymus. Our research provided insights into the molecular regulatory mechanisms that respond to heat-induced OS, and the potential therapeutic implementation of AST-AOX supplementation to mitigate the effects through the modulation of transcription pathways. The complexities of such mechanisms from different tissue types and the varied responses of AOX, whether endogenous or applied, leave us with knowledge gaps that require further research to understand the therapeutic potentials of AST-AOX and investigate the effects of dosing.

## Figures and Tables

**Table 1:** Growth performance indicators of Cobb 500 broiler chickens.

Measurements	TN	HS	HSAX	*p*-Value
BW (g)	2673.68 ^b^ ± 35.71	1848.85 ^a^ ± 61.56	1867.83 ^a^ ± 60.82	0.005
ADFI (g)	94.98 ^b^ ± 1.45	70.68 ^a^ ± 2.38	72.24 ^a^ ± 1.14	0.004
ADG (g)	62.65 ^b^ ± 0.86	42.99 ^a^ ± 1.46	43.45 ^a^ ± 1.44	0.005
FCR	1.52 ^b^ ± 0.02	1.64 ^a^ ± 0.02	1.67 ^a^ ± 0.03	0.005

Letters ^a^ and ^b^ describe significant differences between treatments at *p* < 0.05.

**Table 2:** Genes exhibiting significant differential expression for the thymus tissue of Cobb 500 broilers with *ACTB* as the housekeeping gene for normalization.

Gene	TN	HS	HSAX	*p*-Value
*NFKB1*	1.263 ^a^ ± 0.42	3.168 ^b^ ± 0.45	1.157 ^a^ ± 0.25	0.011
*RELA*	1.153 ^a^ ± 0.27	3.586 ^b^ ± 0.72	2.261 ^a^ ± 0.74	0.012
*IKBKB*	1.257 ^a^ ± 0.38	5.759 ^b^ ± 1.55	3.857 ^a^ ± 0.74	0.009
*NFE2L2*	2.275 ^a^ ± 1.55	8.637 ^b^ ± 3.90	2.582 ^a^ ± 0.46	0.039
*KEAP1*	1.177 ^b^ ± 0.29	4.375 ^a^ ± 1.36	4.675 ^a^ ± 1.28	0.011
*MAF*	1.199 ^a^ ± 0.38	4.330 ^b^ ± 0.72	2.824 ^a^ ± 1.19	0.030
PPARα	1.192 ± 0.28	2.658 ± 0.45	1.791 ± 0.25	0.064
*RXRA*	1.343 ^a^ ± 0.37	6.121 ^b^ ± 1.01	3.148 ^a^ ± 0.63	0.004
*PPARγ*	1.859 ^b^ ± 0.81	25.560 ^a^ ± 9.53	5.452 ^a^ ± 9.84	0.003
*CD36*	1.744 ^a^ ± 0.84	5.811 ^b^ ± 1.37	6.030 ^a^ ± 2.57	0.042
*SOD1*	1.170 ± 0.28	2.098 ± 0.41	2.228 ± 0.39	0.128
*SOD2*	1.070 ± 0.17	1.046 ± 0.29	2.638 ± 0.82	0.049
*SOD3*	1.381 ^a^ ± 0.41	3.994 ^b^ ± 0.64	3.272 ^a^ ± 1.01	0.045
*CAT*	6.447 ± 3.92	12.313 ± 1.47	8.614 ± 2.81	0.291
*GPX1*	1.232 ± 0.33	2.156 ± 0.44	1.862 ± 0.44	0.209
*GPX2*	1.764 ^a^ ± 0.68	6.997 ^b^ ± 1.07	2.392 ^a^ ± 0.52	0.005
*GPX3*	1.266 ± 0.34	2.599 ± 0.36	2.143 ± 0.60	0.085
*GPX4*	1.132 ± 0.25	2.029 ± 0.45	1.578 ± 0.36	0.236
*PRDX1*	1.123 ± 0.25	2.950 ± 0.60	2.224 ± 1.22	0.117
*PRDX3*	1.119 ± 0.23	2.389 ± 0.49	1.540 ± 0.51	0.135
*PRDX4*	1.060 ^a^ ± 0.16	2.601 ^b^ ± 0.43	1.360 ^a^ ± 0.36	0.026
*PRDX6*	1.100 ^a^ ± 0.23	2.888 ^b^ ± 0.33	1.427 ^a^ ± 0.25	0.006
*BCL2*	1.039 ± 0.13	2.479 ± 0.88	1.483 ± 0.41	0.312
*CASP3*	1.043 ^a^ ± 0.12	2.677 b ± 0.48	0.067 ^a^ ± 0.06	0.001
*TP53*	2.976 ± 1.66	4.297 ± 1.40	4.890 ± 2.55	0.567

Letters ^a^ and ^b^ describe significant differences between treatments at *p* < 0.05.

## Data Availability

The original data presented in this study are openly available in Github at https://github.com/sweetiek/Broiler_thymus_astaxanthin.

## Appendix A

**Table A1:** Basal diet ingredients and calculated analysis of broiler diets used in the mixture design for thermal neutral and heat stress including astaxanthin supplement.

Ingredients (%)	Starter	Finisher
Corn	54.86	63.14
Soybean meal	39.49	29.59
Soybean oil	2.00	4.50
Limestone	1.27	0.85
Monocalcium phosphate	0.75	0.50
L-lysine (98–99%)	0.23	0.18
DL-methionine (99%)	0.14	0.12
L-threonine (98–99%)	0.20	0.16
Sodium chloride	0.43	0.35
Sodium bicarbonate	0.12	0.10
Vitamin-mineral premix ^1^	0.50	0.50
Astaxanthin supplement ^2^	0.01	0.01
Total		
Calculated analysis		
ME (kcal/kg)	2909	3203
Soybean meal-CP (%)	22.09	18.07
Calcium (%)	0.75	0.52
Total phosphorus (%)	0.57	0.47
dig phosphorous (%)	0.30	0.23
L-lysine (%)	1.39	1.10
dig L-lysine (%)	1.25	0.99
DL-methionine (%)	0.48	0.41
dig DL-methionine (%)	0.45	0.39
L-cysteine (%)	0.43	0.38
L-threonine (%)	1.03	0.85
dig L-threonine (%)	0.85	0.69
L-tryptophan (%)	0.33	0.26
DL-methionine + L-cysteine (%)	0.91	0.80
L-arginine (%)	1.61	1.31
L-valine (%)	1.22	1.03
L-isoleucine (%)	0.93	0.76
L-leucine (%)	1.89	1.63
Neutral detergent fiber (% DM)	9.13	8.78
Crude fiber (%)	3.97	3.46
Sodium (mg/kg)	0.22	0.18
Chloride (mg/kg)	0.30	0.25
Choline (mg/kg)	1419	1200
Astaxanthin (mg/kg)	---	1.33

Providing the following ^1^ vitamin-mineral premix (per kg of diet): vitamin A (trans-retinyl acetate), 10,000 IU; vitamin D3 (cholecalciferol), 3000 IU; vitamin E (all-rac-tocopherolacetate), 30 mg; vitamin B1, 2 mg; vitamin B2, 8 mg; vitamin B6, 4 mg; vitamin B12 (cyanocobalamin), 0.025 mg; vitamin K3 (bisulphatemenadione complex), 3 mg; choline (choline chloride), 250 mg; nicotinic acid, 60 mg; pantothenic acid (D-calcium pantothenate), 15 mg; folic acid, 1.5 mg; betaíne anhydrous, 80 mg; D-biotin, 0.15 mg; zinc (ZnO), 80 mg; manganese (MnO), 70 mg; iron (FeCO_3_), 60 mg; copper (CuSO_4_·_5_H_2_O), 8 mg; iodine (KI), 2 mg; selenium (Na_2_SeO_3_), 0.2 mg. ^2^ Astaxanthin was mixed with the soybean oil and supplemented in the diet during feed mixing.

**Table A2:** Common components of *Haematococcus* algae (Lorenz; Cyanotech 1999).

	Minimum	Maximum	Mean
Protein	17.30	27.16	23.62
Carbohydrates	36.9	40.0	38.0
Fat	7.14	21.22	13.80
Iron (%)	0.14	1.0	0.73
Moisture	3.0	9.0	6.0
Magnesium (%)	0.85	1.4	1.14
Calcium (%)	0.93	3.3	1.58
Biotin (mg/lb)	0.108	0.665	0.337
L-carnitine (ug/g)	7.0	12	7.5
Folic acid (mg/100 g)	0.936	1.48	1.30
Niacin (mg/lb)	20.2	35.2	29.8
Pantothenic acid (mg/lb)	2.80	10.57	6.14
Vitamin B1 (mg/lb)	<0.050	4.81	2.17
Vitamin B2 (mg/lb)	5.17	9.36	7.67
Vitamin B6 (mg/lb)	0.659	4.5	1.63
Vitamin B12 (mg/lb)	0.381	0.912	0.549
Vitamin C (mg/lb)	6.42	82.7	38.86
Vitamin E (IU/lb)	58.4	333	186.1
Ash	11.07	24.47	17.71

**Table A3:** Gallus gallus oligonucleotide primers used for real-time RT-PCR analysis.

Gene	NCBI Accession No.	Primer Set (5′–3′)
*ACTB*	NM_205518.2	F: 5′-AATTGTGCGTGACATCAAGG
		R: 3′-CACAGGACTCCATACCCAAG
*TBP*	NM_205103.2	F: 5′-GCGGCAGGCTCTGTT
		R: 3′-ACCGAAAAGGTTTTTGACCC
*GAPDH*	NM_204305.2	F: 5′-AAGTCGGAGTCAACGGATTT
		R: 3′-TCACAAGTTTCCCGTTCTCA
*NFKB1*	NM_205134.2	F: 5′-GGACGGCGAAAGGACTCT
		R: 3′-CCATTGCAAACATTTGGGGAT
*RELA*	NM_001396038.1	F: 5’-CGGTTCCGCTATAAGTGTGA
		R: 3’-GTAATGGTTTACGCGGATGG
*IKBKB*	NM_001395965.1	F: 5’-TCCCTGGGAGATGAAGGAG
		R: 3’-TTTGGATGGTTCAGCCTCTT
*NFE2L2*	XM_015287264.4	F: 5′-CAGGGGTAGCAAGGTATGAG
		R: 3′-TGCCTCCAAAGGATGTCAAT
*KEAP1*	GenBank: KU321503.1	F: 5′-GATCGACGGGATGATCTACG
		R: 3′-GGCGTACAGCAGTATGTTCA
*MAF*	NM_001044671.1	F: 5′-CCAGAGTTTTTCATGTACCCG
		R: 3′-CTTTGTAGCTGTCTTCGTGC
*PPARA*	NM_001001464.1	F: 5′-AGCCACTTGCTATCACCAAT
		R: 3′-ACTTAAACTCCTTTATGATTCTGGT
*RXRA*	XM_003642291.6	F: 5′-CTTCCTGCCACTGGATTTCT
		R: 3′-CTGATGACGGAGAAGGGTG
*PPARG*	NM_001001460.2	F: 5′-CTTGACAGCGCCAGAGATTA
		R: 3′-GATTGCACTTTGGCAATCCT
*CD36*	NM_001030731.1	F: 5′-TTTCTTGCAAAGCAGGAGGTT
		R: 3′-CTGATCTTCGTGAGAGAAGCTGTA
*SOD1*	NM_205064.2	F: 5′-AAAAGATGCAGATAGGCACG
		R: 3′-TTATCTCCCCCTCTACCCAG
*SOD2*	NM_204211.2	F: 5′-CCTTCGCAAACTTCAAGGAG
		R: 3′-AGCAATGGAATGAGACCTGT
*SOD3*	XM_040699307.2	F: 5′-CAACTCGCAAACAACGCT
		R: 3′-CTGGTGAGTGAGAACCTGC
*CAT*	NM_001031215.2	F: 5′-TTCCACGTTAAGACCGATCA
		R: 3′-CAATCTTGCCCACTGGAATG
*GPX1*	NM_001277853.3	F: 5′-AATTCGGGCACCAGGAGAA
		R: 3′-CTCGAACATGGTGAAGTTGG
*GPX2*	NM_001277854.3	F: 5′-AGTTCGGCTACCAGGAGAA
		R: 3′-CTTCTGGAACAGGGTGAAGT
*GPX3*	NM_001163232.3	F: 5′-AGGTGAAATGCTACGACTCC
		R: 3′-AGTGCATTCAGTTCGAGGTA
*GPX4*	NM_204220.3	F: 5′-AATGTGCGCTCAGGCG
		R: 3′-AGACGAAGCCCCTGTACT
*PRDX1*	NM_001271932.2	F: 5′-TGCGGGGCTCTTTGTATTAAA
		R: 3′-ATTGCCCATCTGGCATTACA
*PRDX3*	XM_426543.6	F: 5′-CGTTGTCAATGGGGAGTTC
		R: 3′-GGGGCACACAAAGGTGAAAT
*PRDX4*	XM_046912353.1	F: 5′-ATCCCCTTGACTTCACGTTT
		R: 3′-ATCTTCATTGGTCCGAGTCC
*PRDX6*	NM_001039329.3	F: 5′-GACATCAACGCCTACAATGG
		R: 3′-GGCCAAATATGAACACCACA
*BCL2*	NM_205339.3	F: 5′-GAGGATGGGATGCCTTTGTG
		R: 3′-CCACGATAAACTGGGTGACT
*CASP3*	NM_204725.1	F: 5′-GGTGGAGGTGGAGGAGC
		R: 3′-TGAGCGTGGTCCATCTTTTA
*TP53*	NM_205264.1	F: 5′-GTTACCACGACGAGCCACCAA
		R: 3′-TGCAGCGCCTCATTGATCTCCTT

**Table A4:** Gene ontology of differentially expressed genes in the thymus of 6-week-old Cobb 500 broilers exposed to 21 days of heat stress and astaxanthin treatment (*Ensembl*) [42].

Gene	Cellular Component	Molecular Function	Biological Process	Transcript IDs
*NFKB1*	nucleus, cytoplasm, mitochondrion, chromatin	DNA binding transcription factor activity, RNA polymerase II specific DNA binding, chromatin, protein and actinin binding	regulation of transcription by RNA polymerase II, MAPK cascade, JNK cascade, NIK/NF-kappaB signaling	ENSGAL00010005476
*RELA*	cytoplasm	DNA-binding transcription factor activity	regulation of DNA-templated transcription	ENSGALG00010001277
*IKBKB*	cytoplasm, cytosol	protein kinase activity, identical protein binding, protein homodimerization activity, scaffold protein binding, transferrin receptor binding	Protein phosphorylation, I-kappaB kinase/NF-kappaB signaling, cellular response to tumor necrosis factor, regulation of establishment of endothelial barrier, negative regulation of bicellular tight junction assembly	ENSGALG00010017955
*NFE2L2*	nucleus, cytoplasm, Golgi apparatus, chromatin, centrosome, plasma membrane, RNA polymerase II transcription regulator complex	DNA binding transcription factor activity, RNA polymerase II specific DNA-binding transcription factor binding, ubiquitin protein ligase binding	response to oxidative stress, inflammatory response, regulation of gene expression, protein ubiquitination, cell redox homeostasis, positive regulation of glutathione biosynthetic process, regulation of removal of superoxide radicals	ENSGAL00010024107
*KEAP1*	nucleus, cytoplasm, endoplasmic reticulum, Cul3-RING ubiquitin ligase complex, centriolar satellite	RNA polymerase II-specific DNA-binding transcription factor binding, ubiquitin ligase-substrate adaptor activity, disordered domain specific binding, identical protein binding	cellular response to oxidative stress, ubiquitin-dependent protein catabolic process, regulation of DNA-templated transcription, regulation of autophagy	ENSG00000079999
*MAF*	nucleus, cytoplasm, RNA polymerase II transcription regulator complex	DNA-binding transcription factor activity, RNA polymerase II sequence-specific DNA binding	regulation of DNA templated transcription, regulation of transcription by RNA polymerase II, cell development	ENSGALG00010007800
*PPARα*	nucleus, nucleoplasm	RNA polymerase II-specific DNA-binding transcription factor, signaling receptor activity, metal ion binding, lipid binding, ubiquitin conjugating enzyme binding	Negative regulation of transcription by RNA polymerase II, regulation of DNA-templated transcription, negative regulation of reactive oxygen species biosynthesis process, negative regulation of cytokine production involved in inflammatory response, positive regulation of fatty acid oxidation, positive regulation for gluconeogenesis	ENSGALG00010023058
*RXRA*	nucleus, RNA polymerase II transcription regulator complex, receptor complex	RNA polymerase II transcription regulatory region sequence-specific DNA binding, enzyme, peptide, metal ion binding, retinoic acid-responsive element binding, vitamin D response element binding	peroxisome proliferator activated receptor signaling pathway, retinoic acid receptor signaling pathway, positive regulation of Vitamin D receptor signaling pathway, cell differentiation	ENSGALG00010028422
*PPARγ*	nucleus, cytoplasm, intracellular membrane-bounded organelle	DNA-binding transcription factor activity, nuclear receptor activity, zinc and metal ion binding	Regulation of DNA-templated transcription, transcription by RNA polymerase II, intracellular receptor signaling pathway	ENSGALG00010027917
*CD36*	external side of plasma membrane, receptor complex, membrane raft	low-density lipoprotein particle receptor activity, high-density lipoprotein particle binding, Toll-like receptor binding, scavenger receptor activity	MAPK cascade, cell surface receptor signaling pathway, positive regulation of cytosolic calcium ion concentration, nitric oxide mediated signal transduction, intestinal cholesterol absorption, positive regulation of reactive oxygen species metabolic process, lipid transport across blood-brain barrier	ENSGALG000010008392
*SOD2*	mitochondrion	superoxide dismutase activity, oxidoreductase activity, manganese ion binding, metal ion binding, identical protein binding	response to oxidative stress, oxidation-reduction process, negative regulation of oxidative-stress-induced intrinsic apoptotic signaling pathway, response to hydrogen peroxide, removal of superoxide radicals	ENSGALT00000019062
*SOD3*	extracellular space, collagen- containing extracellular matrix	superoxide dismutase activity, copper and metal ion binding	superoxide metabolic process, response to hypoxia	ENSGALG00010009833
*GPX2*	cytosol, intercellular bridge, mitotic spindle	glutathione peroxidase activity, phospholipid-hydroperoxide glutathione peroxidase activity	response to oxidative stress	ENSGALG00010021537
*PRDX4*	cytoplasm, endoplasmic reticulum	antioxidant activity, thioredoxin peroxidase activity, oxidoreductase activity, peroxiredoxin activity, molecular sequestering activity	response to oxidative stress, cell redox homeostasis, hydrogen peroxide catabolic process, reactive oxygen species metabolic process, cellular oxidant detoxification	ENSGALG00010003214
*PRDX6*	nucleus, cytoplasm	antioxidant activity, glutathione peroxidase activity, oxidoreductase activity, peroxiredoxin activity, ubiquitin protein ligase binding	response to oxidative stress, cellular oxidant detoxification, glycerophospholipid catabolic process	ENSGALG00010013528
*CASP3*	nucleus, cytoplasm	endopeptidase activity, hydrolase activity, cyclin-dependent protein serine/threonine kinase inhibitor activity, cysteine-type endopeptidase activity involved in execution phase of apoptosis	T and B cell homeostasis, negative regulation of cytokine production, intrinsic apoptotic signaling pathway, negative regulation of cell cycle, neuron apoptotic process, epithelial cell apoptotic process	ENSGALG00010007067
